# Estimated health and economic impact of quadrivalent HPV (types 6/11/16/18) vaccination in Brazil using a transmission dynamic model

**DOI:** 10.1186/1471-2334-12-250

**Published:** 2012-10-09

**Authors:** Kosuke Kawai, Gabriela Tannus Branco de Araujo, Marcelo Fonseca, Matthew Pillsbury, Puneet K Singhal

**Affiliations:** 1 Temple University, 3307 N. Broad Street, Philadelphia, PA, 19140, USA; 2 Axia.Bio, São Paulo, Brazil; 3 Federal University of São Paulo – UNIFESP, São Paulo, Brazil; 4 Atlas Data Systems, Westfield, NJ, 07090, USA; 5 Merck & Co., Inc., West Point, PA, 19486, USA

## Abstract

**Background:**

Cervical cancer is the second most common cancer among women in Brazil. We examined the health and economic impacts of quadrivalent HPV vaccination in Brazil.

**Methods:**

We adapted a previously developed transmission dynamic model to estimate the effectiveness of HPV vaccination on cervical cancer, cervical intraepithelial neoplasia grades 2 and 3 (CIN2/3), CIN1, and genital warts. We evaluated following vaccination strategies: routine vaccination of 12-year-old girls and routine vaccination in combination with a catch-up vaccination of 12 to 26-year-old women.

**Results:**

The model projected that the vaccination would reduce the incidence rates of HPV 6/11/16/18-related cervical cancer, CIN2/3, CIN1, and female genital warts by 94% to 98% at year 100. Routine vaccination in combination with a catch-up vaccination could prevent approximately 163,000 cases of cervical cancer, 48,000 deaths from cervical cancer, 2.3 million cases of CIN2/3, and 11.4 million genital warts in the next 50 years. The incremental cost-effectiveness ratios for female vaccination strategies ranged from R$350 to R$720 (US$219 to US$450) per quality-adjusted life year (QALY) gained.

**Conclusions:**

Our study demonstrates that quadrivalent HPV female vaccination can be a cost-effective public health intervention that can substantially reduce the burden of cervical diseases and genital warts in Brazil.

## Background

Every year, nearly 530,000 women develop cervical cancer and 275,000 die from the disease worldwide [1]. About 88% of deaths from cervical cancer occur in developing countries [1]. Human papillomavirus (HPV) is the primary cause of cervical cancer, with HPV-types 16 and 18 causing approximately 70% of cases globally [2]. A quadrivalent HPV vaccine against HPV-types 6, 11, 16, and 18 (Gardasil® by Merck & Co., Inc.) was recently developed and is widely available around the world [3-5].

Cervical cancer is the second most common cancer among women in Brazil. The cervical cancer incidence rate is two to three times higher in Brazil (19.2 per 100,000 women every year) than in North America and Europe [6]. The quadrivalent HPV vaccine was approved for use in Brazil to prevent cervical, vulvar, and vaginal cancer, precancerous lesions, and genital warts caused by HPV types 6, 11, 16, and 18. However, HPV vaccination has not been introduced as a national program in Brazil. Understanding the health and economic impacts of HPV vaccination is essential for policy makers to make decisions regarding the introduction of national vaccination programs. Previous cost-effectiveness studies of HPV vaccination in Brazil and other middle-income countries have used a range of different methods [7-11]. Because the mathematical transmission dynamic model is required to describe the transmission of HPV infections and diseases, we used this method to examine the effectiveness of HPV vaccination.

The purpose of our study was to examine the long-term health and economic impacts of quadrivalent HPV vaccination in Brazil using a transmission dynamic model. We evaluated the following vaccination strategies: routine vaccination of 12-year-old girls and routine vaccination of 12-year-old girls in combined with a catch-up vaccination of 12 to 26-year-old women.

## Methods

We adapted a previously developed transmission dynamic model to evaluate the health and economic impacts of HPV vaccination in Brazil [12]. The transmission dynamic model incorporates the direct and indirect benefits (herd immunity) of vaccination. Details of the model have been previously published [12-14]. Here, we briefly described the model structure and methods for adapting the model to Brazil.

### Model structure

The model has demographic and epidemiologic components. The population is divided into sex and 23 age groups to simulate demographic characteristics of the population. Individuals die at sex- and age-specific rates within each group, and individuals are born into the youngest age group at a rate that balances mortality. Each group is further stratified into three sexual activity groups (low, medium, and high rates of sexual partner change). The model accommodates general patterns of mixing between age and sexual activity group. The epidemiologic component of the model simulates heterosexual transmission of HPV-types 6/11/16/18 and progression to cervical intraepithelial neoplasia (CIN) grades 1, 2, and 3, cervical cancer, and genital warts. The population is divided into epidemiologic compartments according to the host's susceptibility to infection or the host's status with respect to infection, immunity, disease, screening, and treatment. All model simulations were conducted in Mathematica® (Wolfram Research, Champaign, IL, USA).

### Model parameters

Model parameters regarding the natural history of HPV 6/11/16/18 infections and diseases, as well as diagnostic characteristics were based on a previous literature review [12]. Assuming that the natural history parameters are similar, we kept the same parameter values used in the U.S. model for Brazil. For model parameters for demographics, sexual behaviors, and clinical management of HPV-related diseases, we conducted a comprehensive literature review to obtain data for Brazil (Table 1) [15-21]. The information collected for clinical management of HPV-related diseases were age-specific cervical cytologic screening and hysterectomy rates and age and stage-specific cervical cancer mortality rates. When data from Brazil were not available, we used data from another country from the same region of the world. Based on the recent literatures, we assumed that approximately 70% of targeted women would receive regular cervical cytologic screening with specific annual screening rates by age (Table 1). We also assumed that that the current screening practice would remain the same throughout the 100 year time horizon.

**Table 1:** Demographic, sexual behavior, cervical cancer screening, and disease model parameters

**Parameter**	**Estimate**		**Source**
**Demographic variables**			
Total population size	203,429,773		[15]
Annual all-cause mortality rate by sex and age	(Data not shown)		[16]
**Sexual behavior variables**			
Annual mean number of sexual partners by sex	Male	Female	[17]
15-19 yrs	1.65	1.29	
20-24 yrs	1.89	1.07	
25-34 yrs	1.40	1.06	
35-44 yrs	1.22	0.98	
45-59 yrs	1.15	0.79	
**Screening variables**			
	Female		
Cervical cancer screening rate, % past 3 years	70		[18-20]
Cervical cancer screening rate, % past year			[18]
<20 yrs	0.0		
20-29 yrs	26.0		
30-34 yrs	30.3		
35-39 yrs	32.6		
40-44 yrs	30.8		
45-49 yrs	30.0		
50-54 yrs	28.6		
55-69 yrs	26.4		
>70 yrs	10.0		
**Disease variables**			
Cervical cancer mortality rates, per year			[21]
Localized cervical cancer			
<40 yrs	0.135		
40-49 yrs	0.066		
50-59 yrs	0.090		
60-69 yrs	0.100		
>70 yrs	0.130		
Regional cervical cancer			
<40 yrs	0.202		
40-49 yrs	0.100		
50-59 yrs	0.136		
60-69 yrs	0.150		
>70 yrs	0.195		
Distant cervical cancer			
<40 yrs	0.808		
40-49 yrs	0.399		
50-59 yrs	0.543		
60-69 yrs	0.602		
>70 yrs	0.780		
Population level hysterectomy rates, % per year			
< 25 yrs	0.01		
25-29 yrs	0.03		
30-34 yrs	0.12		
35-39 yrs	0.36		
40-44 yrs	0.67		
45-54 yrs	0.58		
55-69 yrs	0.20		
>70 yrs	0.20		

### Vaccine strategies and characteristics

We examined the health and economic impacts of three different scenarios: 1) no vaccination, 2) routine vaccination of 12-year-old females, and 3) routine vaccination of 12-year-old females with a catch-up vaccination of 12 to 26-year-old females. Assuming that the national vaccination program will be school-based, routine vaccination coverage was assumed to gradually cover 85% of girls by 12 years of age. We assumed that a catch-up program among women 12 to 26 years of age would gradually increase to cover 95% by 26 years of age. All vaccinated women were assumed to complete a three-dose regimen. We assumed the duration of vaccine protection to be lifelong. We also examined a 20-year duration of protection in a sensitivity analysis. We estimated the cost of vaccination using the Pan American Health Organization (PAHO) vaccine acquisition cost per dose in 2011 (US$15.15 per dose). We assumed a total cost of the three vaccine series to be R$72.72 (US$45.45). The model incorporates the vaccine efficacy from the most recent clinical trials [12].

### Economic data and health utility parameters

Costs were estimated from the perspective of the healthcare system in Brazil. The costs of cytology screening and diagnosis were based on the official value reimbursed by the public health care in Brazil (Table 2) [22]. The costs associated with treatment of genital wart, CIN, and cervical cancer cases are based on published data [7,22]. Quality-adjusted life years (QALYs) were estimated based on health utilities. Because the health utility data were not available from Brazil, we used the same health utility values from the U.S. [12]. Total QALYs were estimated by weighting survival time by the quality of life weights associated with each health state. We examined the cost-effectiveness of introducing HPV vaccination over a time horizon of 100 years. Costs and QALYs were discounted at 3%.

**Table 2:** Cost of diagnosis and treatment of HPV-related diseases

**Parameter**	**R$**	**US$**	**References**
Genital warts-Female	94	59	[22]
Genital warts-Male	114	71	[22]
Cervical cancer screening and visit	17	11	[22]
Colposcopy	3	2	[22]
Biopsy	24	15	[22]
CIN1 episode-of-care	175	109	[22]
CIN2 episode-of-care	534	334	[22]
CIN3 episode of care	534	334	[22]
Localized cervical cancer	7769	4856	[7]
Regional cervical cancer	6520	4075	[7]
Distant cervical cancer	6520	4075	[7]

### Model simulations and validation

Model validation of the natural history component of the U.S. model has been described previously. We assessed the predictive validity of the model by comparing the model outputs and observed epidemiological data regarding cervical cancer incidence and mortality. Age-standardized incidence rate of cervical cancer was 19.2 per 100,000 women per year and cervical cancer mortality rate was 7.3 per 100,000 women per year in Brazil [6,23]. Assuming that 70.7% of cervical cancer is attributable to HPV-types 16 and 18, the HPV16/18-related cervical cancer rate is estimated to be 13.6 per 100,000 women per year and the HPV16/18-related cervical cancer mortality rate is estimated to be 5.2 per 100,000 per year [24]. The model projected an incidence of HPV16/18-related cervical cancer of 13.5 per 100,000 women per year and HPV16/18-related cancer mortality of 5.0 per 100,000 per year. Data regarding the incidence rates of genital warts and CIN were limited [25-27]. The model projected overall incidence of HPV6/11-related genital warts to be 165 per 100,000 per year. The observed overall incidence of genital warts reported in other parts of the world ranged from 100 to 200 episodes per 100,000 per year, with approximately 90% of cases attributable to HPV6/11 [28-30].

### Sensitivity analysis

The previous analysis in the U.S model has identified the most influential parameters [12]. Based on the previous findings, we conducted a one-way sensitivity analysis on parameters that incorporates different values for duration of vaccine protection, costs of vaccine series, vaccine coverage rates, HPV-related disease cost, and discounting. We also examined a scenario assuming no quality of life adjustments. In addition, we examined a scenario assuming no effect of HPV-types 6 and 11. Finally, we examined a pessimistic scenario that assumed 20 years of vaccine protection, low HPV-related disease cost (decreased by 25%), and high health utility values (0.97 for HPV-related disease).

## Results

### Health impact of HPV vaccination

**Figure 1:**
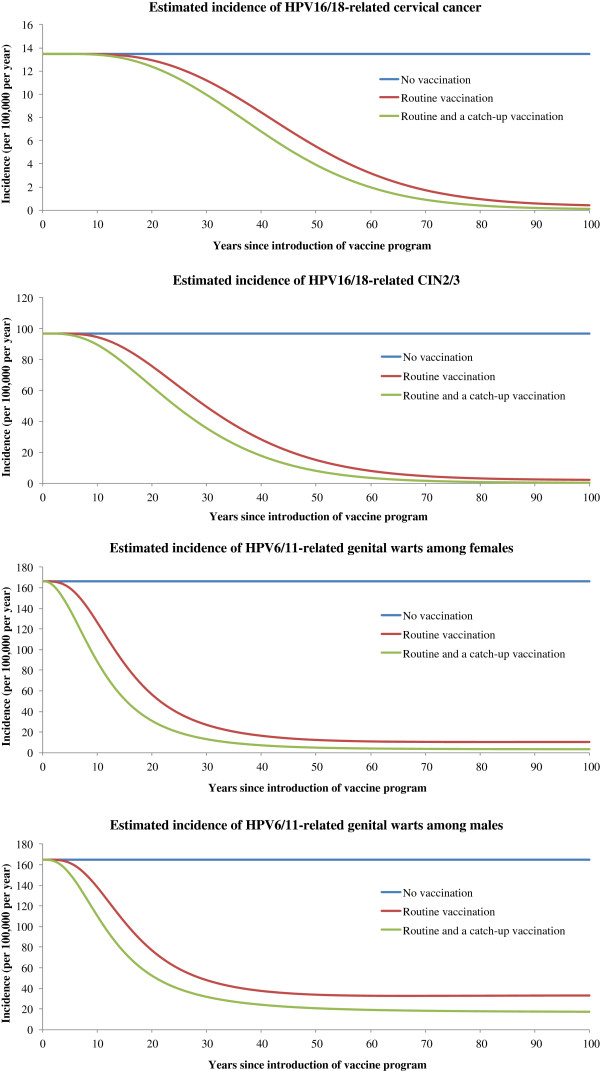
**The incidence rates of HPV16/18-related cervical cancer, HPV16/18-related CIN2/3, and HPV6/11-related genital warts among females and males by vaccination strategy during the next 100 years.** We assumed that approximately 71% of cervical cancer cases were attributable to HPV 16 and 18, and 90% of genital warts cases were attributable to HPV 6 and 11 in Brazil.

The model projected that routine quadrivalent HPV vaccination of 12-year-old girls will reduce the incidence rate of HPV16/18-related cervical cancer by 59% at year 50 and by 97% at year 100 (Figure 1). The HPV16/18-related cervical cancer mortality rate was also projected to decline by 97% at year 100. The routine vaccination of 12-year-old girls in combination with a catch-up vaccination of 12 to 26-year-old women will achieve greater and earlier reduction in the number of cumulative cases and deaths from cervical cancer than the routine vaccination. Routine vaccination in combination with a catch-up vaccination will reduce the incidence rate of HPV16/18-related cervical cancer by 71% at year 50 and by 99% at year 100. We estimated that routine vaccination in combination with a catch-up vaccination could prevent 162,769 cumulative cases of cervical cancer by year 50 and 795,693 cases by year 100 (Table 3). Routine vaccination in combination with a catch-up vaccination could prevent 47,802 cumulative deaths from cervical cancer by year 50 and 278,283 deaths by year 100.

**Table 3:** Estimated cumulative cases of HPV-types 6/11/16/18-related disease prevented with routine vaccination of 12 year-old girls or routine vaccination combined with a catch-up vaccination of 12- to 26-year-old women in Brazil

**HPV-related diseases**	**Routine vaccination**				**Routine and a catch-up vaccination**			
	**10 years**	**25 years**	**50 years**	**100 years**	**10 years**	**25 years**	**50 years**	**100 years**
Cervical cancer	22	6,449	118,825	712,067	117	12,954	162,769	795,693
Cervical cancer deaths	3	1,296	33,698	247,572	15	2,700	47,802	278,283
CIN1	3,797	132,541	763,332	2,437,178	14,744	218,133	943,965	2,674,632
CIN2/3	4,412	250,896	1,829,778	6,498,426	18,948	435,052	2,301,481	7,135,912
Genital warts among women	117,260	1,523,777	5,235,130	13,151,591	318,735	2,180,661	6,177,401	14,459,056
Genital warts among men	70,649	1,173,031	4,314,599	11,031,658	189,698	1,691,626	5,199,269	12,650,796

Our model projected that the HPV vaccination program will reduce the incidence rates of genital warts among women and men, and CIN2/3 before it reduces the incidence rate of cervical cancer (Figure 1). The model projected that routine vaccination of 12-year-old girls will reduce the incidence of HPV6/11-related genital warts among females by 94%, HPV6/11/16/18-related CIN1 by 98%, and HPV16/18-related CIN2/3 by 98% at year 100. Through reducing HPV6/11 infections, routine female vaccination could also provide indirect benefits to men by reducing the incidence of genital warts by 70% at year 100. Compared to routine vaccination, routine vaccination in combination with a catch-up vaccination could result in earlier and greater reduction in the cumulative cases of genital warts, CIN1, and CIN2/3 (Table 3). Routine vaccination in combination with a catch-up vaccination could avoid 508,433 cumulative cases of genital warts among women and men in the first 10 years and 3,872,287 cumulative cases in the first 25 years.

### Economic impact of HPV vaccination

Routine vaccination program could avoid a total cost of approximately R$1.74 billion (US$1.09 billion) from HPV6/11/16/18-related diseases in the next 100 years, while routine vaccination in combination with a catch-up vaccination program could avoid R$2.10 billion (US$1.31 billion) in the next 100 years (discounted at 3%). We estimated that about 67.3% of the total avoided costs from routine and a catch-up vaccination program would be from cervical diseases and 32.7% would be from genital warts.

The incremental cost-effectiveness ratio (ICER) for routine vaccination of 12-year-old girls’ strategy compared to no vaccination strategy was R$350 per QALY gained (US$219 per QALY gained; Table 4). Routine vaccination in combination with a catch-up vaccination of 12 to 26-year-old women was a more effective strategy than the routine vaccination, with an ICER of R$720 per QALY gained (US$450 per QALY gained).

**Table 4:** Cost-effectiveness analysis of HPV types 6/11/16/18 vaccination in Brazil

**Strategies**^**1**^	**Discounted total**			**Incremental**		
	**Costs (R$) /person**	**QALYs /person**	**Costs (R$) /person**	**QALYs /person**	**Costs (R$) /QALYs**	**Costs (US$) /QALYs**
No vaccination	90.64	27.03841	-	-	-	-
Routine vaccination	94.38	27.04912	3.74	0.01071	350	219
Routine and a catch-up vaccination	96.09	27.05150	1.71	0.00238	720	450

^1^ Strategies are listed in order of increasing effectiveness.

We also conducted sensitivity analyses (Table 5). Assuming no quality of life benefits, the ICER for routine vaccination was R$404 (US$253) per year of life saved and adding a catch-up vaccination was R$804 (US$503) per year of life saved. When the benefits of preventing HPV16/18-related cervical diseases were considered alone, the ICER for routine vaccination was R$717 (US$448) per QALY and adding a catch-up vaccination was R$1117 (US$698) per QALY. When the assumed duration of vaccine protection was reduced from lifelong to 20 years, the ICER for routine and a catch-up vaccination strategy was R$1049 (US$656) per QALY gained compared to no vaccination. Cost of vaccines and discounting rate also influenced the results. Finally, when we examined the pessimistic scenario, we found that ICER comparing routine and a catch-up vaccination versus no vaccination was R$1596 (US$998) per QALY gained.

**Table 5:** Incremental cost-effectiveness ratio (ICER) for sensitivity analyses

**Parameter**	**Routine vaccination**	**Routine and a catch-up vaccination**
	**US$ per QALYs**	**US$ per QALYs**
Base case	219	450
Cost per year of life saved (no quality of life adjustments)	253	503
HPV16/18-related cervical diseases only	448	698
Duration of vaccine protection = 20 years	Dominated^1^	656
Vaccine coverage= 50% for routine and 60% for catch-up	106	250
Vaccine coverage= 95% for routine and 98% for catch-up	258	425
High cost of vaccine series (increased by 25%)	406	686
High cost of treating HPV-related disease (increased by 25%)	86	326
Low cost of treating HPV-related disease (decreased by 25%)	350	573
Discounted at 0%	Cost saving	Cost saving
Discounted at 5%	900	1201
Pessimistic scenario^2^	Dominated^1^	998

^1^ A strategy is “weakly” dominated if a more effective strategy has a lower incremental cost-effectiveness ratio.

^2^ Pessimistic scenario assumes low HPV-related disease cost (decreased by 25%), 20 years of vaccine protection, and high health utilities (0.97 for HPV-related disease).

## Discussion

Our study demonstrates that quadrivalent HPV female vaccination can substantially reduce the burden of cervical diseases and genital warts in Brazil. The major advantage of our analytic approach is the use of transmission dynamic model that incorporates the direct and indirect benefits of vaccination while evaluating effectiveness at the population level over time. We found that the routine vaccination of 12-year-old girls in combination with a catch-up vaccination of 12 to 26-year-old women can be a cost-effective strategy that can achieve earlier and greater reduction in HPV-related diseases than the routine vaccination. Routine vaccination in combination with a catch-up vaccination could prevent approximately 163,000 cases of cervical cancer, 48,000 deaths from cervical cancer, 2.3 million cases of CIN2/3, and 11.4 million genital warts in the next 50 years.

The World Health Organization considers an intervention to be "very cost-effective" when its incremental cost-effectiveness ratio is below GDP per capita [31]. We found that the incremental cost-effectiveness ratios for HPV vaccination strategies ranged from US$219 to US$450 per QALY gained, which fell below a GDP per capita (US$10,710 in Brazil). In order to address uncertainty of the parameters, we conducted extensive one-way sensitivity analyses. For an example, duration of protection remains uncertain. Even when we decreased the duration of vaccine protection to 20 years, we found that the HPV vaccination was cost-effective especially the vaccination strategy that included a catch-up vaccination. Four prior studies examined the cost-effectiveness of HPV vaccination in Brazil [7-10]. Goldie et al. (2007) used individual-based stochastic models and showed that routine vaccination would cost I$120 to I$820 per year of life saved [7]. Kim et al. (2007) also found vaccination to be cost-effective using dynamic models [8]. Using the similar individual-based dynamic model by Kim et al., Vanni et al. (2012) recently found that the ICER for the quadrivalent HPV vaccination that incorporated the benefits of preventing genital warts to be US$255/QALY assuming similar cost of vaccination as ours ($55 for a total cost of vaccinating woman) with discounting at 5% [10]. Colantonio et al. (2009) used Markov models and found the ICER to be US$10,200 per QALY assuming US$210 for a total cost for vaccinating woman [9]. Higher ICER results may be because Colantonio et al. (2009) assumed higher vaccine cost than ours and used cohort model that did not take into account of herd immunity. In spite of differences in the model structure and assumptions about model parameters regarding natural history of HPV disease, vaccine property, health utilities, and costs, all studies consistently found HPV vaccination of females to be cost-effective in Brazil.

Contrary to most previous studies, we incorporated the potential impact of vaccination on HPV6/11-related genital warts. The quadrivalent HPV vaccine was projected to reduce the incidence of genital warts in a short period of time. This is consistent with a rapid decline in the incidence of genital warts observed among young women in Australia where vaccination has been already implemented [32,33]. Although numerous studies from North America and Europe have shown that HPV female vaccination is generally cost-effective, our incremental cost-effectiveness ratios in Brazil were lower than that in those countries [34-36]. Despite the low cost of treatment for HPV-related diseases, quadrivalent HPV vaccination in Brazil can be a cost-effective intervention, because it can prevent substantial burden of cervical cancer and genital warts.

Our model projected that HPV vaccination could prevent approximately 163,000 cases of cervical cancer, 48,000 deaths from cervical cancer and 11.4 million genital warts in the next 50 years. Many women who suffer from cervical cancer are young and actively caring for their families and it could have devastating consequences to their children and families who lose their mothers. Because we cannot incorporate such effects in cost-effectiveness analysis, our results likely underestimate the potential societal benefits of vaccination. Moreover, our model projected the substantial impact of HPV vaccination on genital warts, which may have long-term psychological and/or physical consequences and profoundly affect patient's quality of life [29,37]. For example, even after treatment, women and men may experience anxiety of recurrence or persistence of genital warts [29]. Reducing cases of genital warts will not only reduce healthcare utilization but will also free up resources for diagnosis and treatment of other diseases.

Our study has several limitations. Although the model was built on available current knowledge on HPV diseases, more studies are needed to understand the natural history of HPV. Detailed data specific to Brazil, such as healthcare seeking behaviors and age- and stage-specific mortality rates from cervical cancer, were limited. Because health utility data were not available from Brazil, we used the health utility values from the U.S. Our model does not incorporate demographic changes in the future, such as population growth. Our model did not account for any temporal changes in screening practice or possible introduction of new screening methods such as HPV DNA testing. As some researchers have examined, it is important to consider screening changes in future research [38,39]. Although the coverage rate of HPV vaccination is unknown, we assumed high coverage because Brazil has a strong national immunization program that has achieved high (>90%) immunization coverage in many currently scheduled vaccines [40].

We only assessed fit of the adapted model by assessing overall incidence of cervical cancer and did not assess age-specific data. We did not employ calibration method that identifies parameters that best predict observed data. Moreover, because of the complexity of the model, it was not feasible to conduct probabilistic sensitivity analysis.

Our projected incidence rates of HPV6/11-related genital warts were comparable to the previously reported incidence rates in other parts of the world [28-30]. A survey conducted by the Ministry of Health in Brazil found that 5.7% of pregnant women reported having a history of clinical diagnosis of genital warts [25]. However, data regarding the incidence of genital warts in the general population in Brazil were not available.

HPV vaccine may have cross-protection against non-vaccine, oncogenic HPV-types; however, the duration and efficacy of the cross-protection remains uncertain [41]. If this additional benefit of vaccine was considered, we would have more favorable cost-effectiveness. We did not consider the potential benefits of HPV vaccination on other HPV-related diseases such as vulvar, vaginal, anal, head and neck, and penile cancers, and recurrent respiratory papillomatoses. Previous studies in the U.S. included these diseases and showed an improvement in incremental cost-effectiveness ratios [12,42]. The HPV vaccination of males could also be important because it may prevent some of these cancers and genital warts.

## Conclusion

In conclusion, our study demonstrates that quadrivalent HPV vaccination can substantially reduce the burden of cervical diseases and genital warts in Brazil. Our model results show that HPV vaccination of females, particularly routine vaccination of 12-year-old girls in combination of a catch-up vaccination of 12 to 26-year-old women, can be a cost-effective intervention in Brazil.

## Competing interests

This study was supported by Merck & Co., Inc. KK is a post-doctoral fellow funded by Merck & Co., Inc. MP is a consultant working with Merck & Co., Inc. PKS is employed by Merck & Co., Inc.

## Authors’ contributions

KK, GTBA, and MF collected data. KK, MP, and PKS performed the analyses. KK drafted the manuscript. All authors reviewed and approved the final version of the manuscript.

## Pre-publication history

The pre-publication history for this paper can be accessed here:

http://www.biomedcentral.com/1471-2334/12/250/prepub
